# Chicken Infectious Anemia Virus Markedly Enhances the Pathogenicity of Infectious Bronchitis Virus–Infected Chickens

**DOI:** 10.1155/tbed/2499058

**Published:** 2026-04-17

**Authors:** Hao Chen, Shuangshuang Ma, Liuyang Yuan, Hongchun Yang, Shijun Xu, Shufeng Feng, Yaping Wang, Aobo Xu, Xiaoran Xuan, Ziqi Ling, Yang Li, Hongbin Si, Gonghe Li, Changbo Ou

**Affiliations:** ^1^ College of Animal Science and Technology, Guangxi University, Nanning, 530004, China, gxu.edu.cn; ^2^ China Animal Health and Epidemiology Center, Qingdao, 266032, China; ^3^ Guangxi Zhuang Autonomous Region Engineering Research Center of Veterinary Biologics, Nanning, 530004, China; ^4^ Guangxi Key Laboratory of Animal Reproduction, Breeding and Disease Control, Nanning, 530004, China

**Keywords:** chicken infectious anemia virus, co-infection, immunosuppression, infectious bronchitis virus, pathogenicity

## Abstract

Chicken infectious anemia virus (CIAV) and infectious bronchitis virus (IBV) represent two of the most economically significant pathogens in the global poultry production. In recent years, coinfections involving CIAV and IBV have become increasingly prevalent in clinical scenarios across Southern China and globally. Nevertheless, the underlying synergistic pathogenic mechanisms remain largely elusive. This study establishes a coinfection model using specific pathogen‐free (SPF) chickens infected with field‐derived strains CIAV GDHY230813 and IBV CK/CK/GX/LA/071423 to systematically investigate pathogenic interactions. Our findings reveal a significant synergistic pathogenic effect through multiple experimental parameters between CIAV and IBV. Compared to the monoinfected controls, coinfected SPF chickens exhibited marked clinical deterioration: Survival rates decreased from 93.5% (CIAV) and 96% (IBV) to 76%, accompanied by profound growth retardation. In terms of viral transmission, coinfection markedly increased the viral shedding of both viruses through the respiratory and digestive tracts. At 14 days postinfection (dpi), the viral detection rates in both oropharyngeal and cloacal swabs soared to 100% in the coinfected group, up from 75% in the corresponding monoinfected groups. Moreover, coinfection facilitated the replication of IBV in the trachea, lungs, kidneys, and cecal tonsils. However, it did not lead to a significant change in the replication levels of CIAV. Pathological examinations revealed more severe lesions in the coinfected group, encompassing exacerbated atrophy of the thymus and bursa of Fabricius, splenomegaly, and heightened infiltration of inflammatory cells in various tissues. In summary, our findings suggest that coinfection with CIAV and IBV results in significant synergistic pathogenicity, worsening the severity of clinical disease and amplifying the potential for viral transmission. This study offers a vital theoretical foundation for comprehending the interactions between these two viruses and highlights the imperative of implementing integrated control strategies against both CIAV and IBV in chickens.

## 1. Introduction

Chicken infectious anemia (CIA), caused by the nonenveloped, single‐stranded circular DNA virus CIA virus (CIAV; genus Gyrovirus, family Anelloviridae), represents a globally significant immunosuppressive disease in poultry production [1, 2]. First identified in Japan in 1979, CIAV has since achieved a global distribution, emerging as a pathogen of substantial economic concern in poultry production, with a notably high prevalence in China. The virus exhibits broad host tropism, infecting chickens across age groups and breeds through both vertical and horizontal transmission routes [3]. Clinically, CIAV induces severe aplastic anemia and generalized immunosuppression, characterized by thymic and bursal atrophy, bone marrow hypoplasia, and multiorgan lesions [4, 5]. Pathophysiological manifestations include lethargy, ruffled feathers, growth retardation, and weight loss, accompanied by quantitative and functional deficiencies in T lymphocytes that compromise host immune competence and predispose to secondary infections [6]. Adult chickens are primarily infected through horizontal transmission and often remain subclinical, yet they persistently shed the virus into the environment. Critically, vertical transmission represents the most significant route of spread. As demonstrated by Cardona et al., CIAV can persist long‐term in reproductive tissues (ovaries and vas deferens) of infected breeders and be transmitted vertically through mating, resulting in infected offspring embryos [7]. Chicks infected via this route typically develop severe clinical disease and, due to the resulting immunosuppressed state, exhibit high susceptibility to secondary infections by other pathogens. Epidemiological data from an extensive nationwide survey (2017–2019) revealed that CIAV not only had the highest detection rate among all monitored pathogens but was also most frequently identified in coinfections with other viruses, including reticuloendotheliosis virus (REV), infectious bursal disease virus (IBDV), and fowl adenovirus (FAdV) [8]. Recent studies further indicate a sustained increase in CIAV prevalence, with detection rates surpassing 80% in several regions [9, 10]. Currently, a primary strategy to control CIAV spread involves vaccinating breeder chickens to induce high levels of maternal antibodies, thereby providing early passive immune protection to their chicks. However, despite extensive research on various vaccine platforms (including inactivated, subunit, vector vaccine, and attenuated vaccines), no officially approved commercial CIAV vaccine is available in China [11–14]. Consequently, effective prevention and control of vertical transmission remain significant challenges for the poultry industry. Compounding the challenge, the host range of CIAV extends beyond chickens to include quails, pigeons, and other avian species, with environmental persistence also documented [15], suggesting potential for interspecies transmission that complicates global control strategies.

Infectious bronchitis virus (IBV), first reported in 1931, is another major pathogen responsible for acute, highly contagious respiratory disease in poultry worldwide. As a gamma‐coronavirus, its high genetic diversity stems from the error‐prone RNA‐dependent RNA polymerase and frequent recombination events, leading to the continuous emergence of new genotypes and serotypes [16, 17]. Based on the S1 gene sequence, IBV is currently classified into 10 genotypes comprising 41 lineages [18]. Pathotypically, IBV strains demonstrate tissue tropism‐based differentiation into respiratory, nephropathogenic, enterotropic, and proventriculotropic strains [19]. A critical challenge is the poor cross‐protection among different serotypes [20], which, combined with immune pressure from extensive vaccination, often leads to vaccine failures and the persistent circulation of novel variants, making control exceedingly difficult [21]. In China, since its initial detection in 1996, IBV has undergone explosive diversification, with at least 10 genotypes (GI‐7, GI‐13, GI‐19, GI‐22, GI‐25, GI‐28, GI‐29, GIII‐1, GVI‐1, and GVII‐1) cocirculating in commercial poultry [22, 23]. Among these, GI‐19 and GI‐13 have emerged as dominant epidemic strains, not only demonstrating widespread prevalence but also a high propensity for recombination [19]. Notably, the GI‐19 genotype has become a predominant threat in many countries, posing a continuous risk to global poultry farming [24].

In practical poultry production, mixed infections occur far more frequently than single‐pathogen infections [25–27]. When hosts are coinfected with multiple pathogens either simultaneously or sequentially, complex pathogen–pathogen interactions frequently manifest, resulting in atypical clinical presentations that substantially complicate disease diagnosis and control [28, 29]. This phenomenon is particularly pronounced in cases involving immunosuppressive viruses. Previous research has demonstrated that CIAV coinfection with the novel gyrovirus homsa 1 (GyH1) induces more profound immunosuppression [30]. A synergistic enhancement of viral replication was reported during coinfection between Marek’s disease virus (MDV) and avian leukosis virus subgroup J (ALV‐J), leading to exacerbated pathology and increased mortality [31]. Similarly, a synergistic interaction between ALV‐J and CIAV has been confirmed to significantly enhance overall pathogenicity in chickens [32]. As a prototypical immunosuppressive pathogen, CIAV not only causes direct damage to the immune system but also compromises vaccine efficacy, thereby facilitating secondary infections and markedly enhancing the virulence of coinfecting pathogens [8].

Despite the frequent detection of CIAV and IBV coinfections in Southern Chinese poultry flocks, as revealed by our epidemiological surveillance, the pathogenic interplay between these two viruses remains poorly characterized. Field observations of elevated mortality and exacerbated lesions in coinfected birds strongly suggest a synergistic effect, yet this hypothesis lacks experimental validation. While the consequences of CIAV coinfection with other agents like ALV and MDV have been studied, a systematic investigation into CIAV–IBV copathogenicity is lacking. To address this gap, we established a sequential infection model in specific pathogen‐free (SPF) chickens using the southern Chinese isolates CIAV GDHY230813 and IBV CK/CH/GX/LA/071423. In this model, birds were infected with CIAV at 1 day old, followed by IBV at 7 days old. This design aimed to reproduce a common field scenario: secondary IBV challenge in chicks that are already immunosuppressed due to vertical or early CIAV infection. This study is designed to comprehensively assess the effects on host pathogenicity, immune responses, and viral replication kinetics. Our work will elucidate the mechanisms underlying their synergy and provide a scientific basis for developing targeted intervention measures.

## 2. Materials and Methods

### 2.1. Virus and Animals

The CIAV strain GDHY230813 (GenBank: PX496712) and IBV strain CK/CH/GX/LA/071423 (GenBank: PP516426.1) used in this study were originally isolated from clinical samples collected in Southern China during 2023 [19]. Both viruses underwent three serial passages in SPF chickens and were subsequently stored at −80°C in our laboratory’s virus repository. For experimental infections, 1‐day‐old SPF chicks were obtained from fertile eggs sourced from Boehringer Ingelheim Vital Biotechnology Co., Ltd. (Beijing, China). The eggs were incubated under standardized conditions (37.8°C, 55% relative humidity) in our laboratory’s hatching facility.

### 2.2. Animal Infection and Sampling

A total of 100 1‐day‐old SPF chicks were randomly assigned to four experimental groups (*n* = 25 per group), as detailed in Table 1. Chicks in the CIAV monoinfection group were inoculated intramuscularly in the femoral muscle with 1 × 10^6^ copies of the CIAV strain GDHY230813 in 0.2 mL of phosphate‐buffered saline (PBS). Chicks in the IBV monoinfected group were inoculated 10^5^ EID_50_ of the IBV strain CK/CH/GX/LA/071423 in 0.2 mL PBS through intraocular and intranasal routes. The coinfection group received CIAV (as above) at day 1 posthatch, followed by IBV (as above) at day 7 posthatch (Figure 1A). Chicks in the negative control group received 0.2 mL PBS via corresponding routes at identical time points. All chickens were housed in isolators under standard conditions and provided sufficient water and feed. Clinical signs and mortality were recorded daily for 28 days postinfection (dpi). On 14 and 21 days postinfection, five chickens from each group were randomly selected and humanely euthanized for sample collection. At the age of 14 and 21 days for chicks, five chickens per group were randomly selected for sample collection. The collected samples included oropharyngeal and cloacal swabs, blood, and tissues from the thymus, trachea, spleen, lung, liver, kidney, bursa of Fabricius, and cecal tonsils. These samples were subsequently processed for histopathological examination and viral load quantification. The overall experimental timeline is schematically represented in Figure 1A.

Figure 1Experimental design and effects of CIAV and IBV coinfection on growth performance. (A) Schematic diagram of the animal challenge experimental timeline. (B) Body weight changes of chickens in different groups from day 1 to day 21 presented as a percentage of the initial body weight (value at 1 day of age set as 100%). (C) Survival curve of chickens in different groups. Data are shown as mean ± SD.

**Figure:**
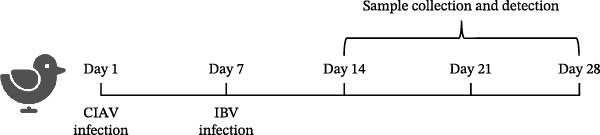
(A)

**Figure:**
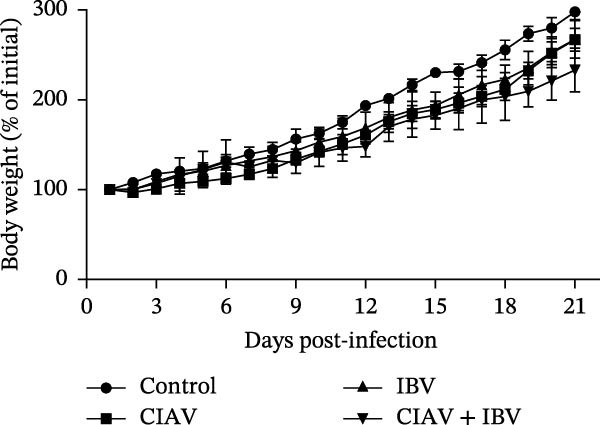
(B)

**Figure:**
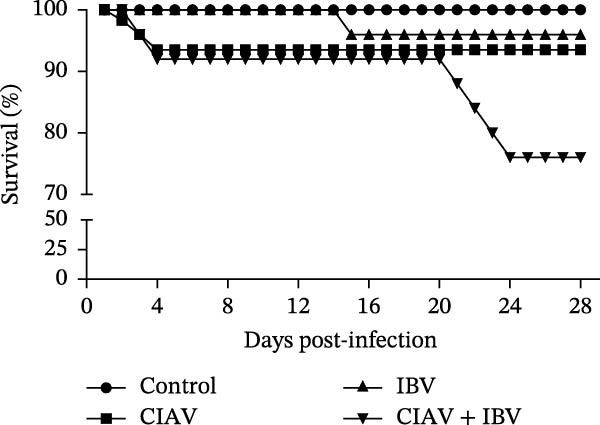
(C)

**Table 1:** Experimental design for CIAV and IBV coinfection in chickens.

Groups	Number of chickens	Viral challenge doses
CIAV	25	200 μL containing 10^6^ copies of CIAV
IBV	25	200 μL containing 10^5^ EID_50_ of IBV
CIAV + IBV	25	200 μL of mixed viral solution containing 10^5^ EID_50_ of IBV and 10^6^ copies of CIAV
Mock	25	200 μL sterile saline

### 2.3. Observation of Main Pathogenic Indicators

Key pathogenic indicators were monitored from day 1–28 postinfection (dpi). Body weights were recorded daily to generate growth curves. Body weight data are presented as the percentage change from the initial weight to allow for better comparison across groups. Clinical signs and mortality were monitored and recorded daily postchallenge, and survival rates were calculated accordingly. Mortality refers specifically to spontaneous deaths, and any birds that reached predefined humane endpoints were euthanized and excluded from the analysis. At 14 and 21 days postinfection (dpi), five chickens per group were randomly selected for sample collection.

Anticoagulated blood samples were obtained from the wing vein and analyzed using a BH‐5190vet automated hematology analyzer (URIT, China) to determine the counts of white blood cells (WBCs) and red blood cells (RBCs), as well as hematocrit (HCT) levels. Simultaneously, the thymus, bursa of Fabricius, and spleen were excised and weighed. The immune organ index was calculated to evaluate the impact of viral infection on immune organ development using the following formula: Immune Organ Index (mg/g) = Immune Organ Weight (mg)/Body Weight (g).

### 2.4. Viral Load Quantification by qPCR

Viral DNA/RNA was extracted from respective tissues to quantify CIAV and IBV viral loads using SYBR Green I real‐time PCR. Specific primers for CIAV and IBV were designed based on the VP1 gene of the Cux‐1 strain (GenBank: M55918.1) and the N gene as specified in the national standard for avian infectious bronchitis diagnostics (GB/T 23197‐2022), respectively. All primers (sequences listed in Table 2) were synthesized by Sangon Biotech (Shanghai, China). Quantitative PCR was performed using the ChamQ Universal SYBR qPCR Master Mix (Vazyme, Nanjing, China) on a LightCycler 480 Instrument II (Roche, Switzerland). The thermal cycling protocol consisted of an initial denaturation at 95°C for 30 s, followed by 40 cycles of 95°C for 10 s and 60°C for 30 s.

**Table 2:** The primers for real‐time qPCR.

Primer	Sequence (5′‐3′)
CIAV	GCAGGGGCAAGTAATTTCAA
CIAV	GCCACACAGCGATAGAGTGA
IBV‐F	TTGAAGGTAGYGGYGTTCCTGA
IBV‐R	CAGMAACCCACACTATACCATC
β‐Actin‐F	TGTGCGTGACATCAAGGAGAAG
β‐Actin‐R	TACCACAGGACTCCATACCCAAG

Total RNA was extracted from trachea, lung, kidney, and cecal tonsils using VeZol Reagent (Vazyme, Nanjing, China). Complementary DNA (cDNA) was synthesized using the HiScript III RT SuperMix for qPCR (Vazyme, Nanjing, China), according to the manufacturer’s instructions. The relative expression level of the IBV N gene was calculated using the 2^^–ΔΔCT^ method, with the *β*‐actin gene serving as the endogenous control [19]. Total DNA was extracted from blood, thymus, spleen, bursa of Fabricius, and liver using the TIANamp Genomic DNA Kit (TIANGEN, Beijing, China). The absolute viral DNA copy number was determined by qPCR using a standard curve generated from serially diluted pMD18T‐VP1 plasmid.

### 2.5. Histopathological Examination

For histopathological examination, tissue samples, including the thymus, spleen, liver, bursa of Fabricius, trachea, lung, and kidney, were collected and immediately fixed in 10% neutral buffered formalin. The fixed tissues were then processed through standard dehydration and clearing procedures, embedded in paraffin, and sectioned. The sections were stained with hematoxylin and eosin (H&E) and examined under a light microscope to evaluate the pathological lesions induced by CIAV and IBV coinfection.

### 2.6. Statistical Analysis

All data are expressed as mean ± SD and were analyzed using SPSS (version 27.0) and GraphPad Prism (version 10.1.2) software. The significance of differences between groups was assessed by one‐way ANOVA or Student’s *t*‐test, as appropriate. Significance levels are indicated as follows: *p*  > 0.05 (ns), *p*  < 0.05 ( ^∗^), *p*  < 0.01 ( ^∗∗^), and *p*  < 0.001 ( ^∗∗∗^).

## 3. Results

### 3.1. CIAV and IBV Coinfection Lowers Growth Performance and Survival

The impact of viral infection on body weight gain is shown in Figure 1B. During the initial stage (1–7 days postinfection, dpi), no significant differences in body weight were observed among most groups, except for the CIAV monoinfected group, which exhibited a significantly lower body weight compared to the control and IBV‐infected groups. From 14 dpi onwards, significant growth retardation became apparent in all infected groups, with body weights markedly lower than those in the control group. Notably, the coinfected group consistently demonstrated the most severe growth suppression, and its body weight became significantly lower than the IBV monoinfected group by 21 dpi. Viral challenge also affected survival rates (Figure 1C). The control group maintained 100% survival throughout the study period. In contrast, the CIAV and IBV monoinfected groups showed survival rates of 93.5% and 96%, respectively. The coinfected group demonstrated the highest mortality, with a survival rate of only 76%. These results collectively indicate that coinfection with CIAV and IBV results in more severe growth retardation and increased mortality compared to monoinfections, indicating potential synergistic pathogenic effects between these two viruses.

### 3.2. Coinfection With CIAV and IBV Leads to Thymic Atrophy in Comparison to IBV Monoinfection

The impact of CIAV and IBV infection on the development of immune organs was assessed by measuring the organ indices of the thymus, spleen, and bursa of Fabricius at 14 and 21 days postinfection (dpi) (Figure 2). The thymus index was significantly reduced in both the CIAV monoinfected and coinfected groups compared to the control and IBV monoinfected groups at both time points. In contrast, the thymus index of the IBV monoinfected group showed no significant difference from the control group. Conversely, the spleen index was significantly elevated in the CIAV monoinfected and coinfected groups relative to the control and IBV monoinfected groups. Although no significant differences were observed in the bursa of Fabricius index, a notable trend indicated that IBV monoinfection might induce bursal swelling, whereas coinfection appeared to exacerbate bursal atrophy compared to the CIAV monoinfected group. Collectively, these findings demonstrate that while IBV monoinfection alone has minimal impact on immune organ indices, coinfection with CIAV synergistically exacerbates thymic atrophy and may promote bursal atrophy, suggesting a combined detrimental effect on the development of immune organs.

Figure 2Effect of coinfection on the immune organ index of SPF chickens. (A) Thymus index. (B) Spleen index. (C) Bursa of Fabricius index. At 14 and 21 days of age, five SPF chickens were randomly selected from each group, and thymus, spleen, and bursa of Fabricius were collected to calculate the immune organ index. Immune organ index = Immune organ weight (mg)/total chicken weight (g). Data are shown as mean ± SD. Significant differences between groups were determined by one‐way ANOVA.  ^∗^ *p* < 0.05 (significant);  ^∗∗^ *p* < 0.01 (highly significant).

**Figure:**
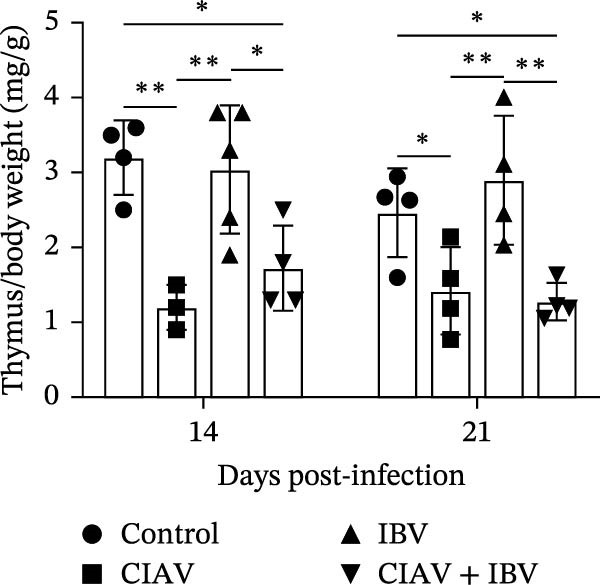
(A)

**Figure:**
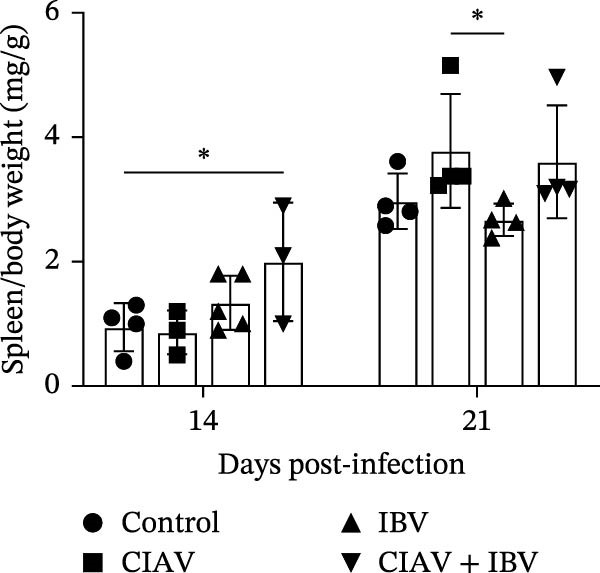
(B)

**Figure:**
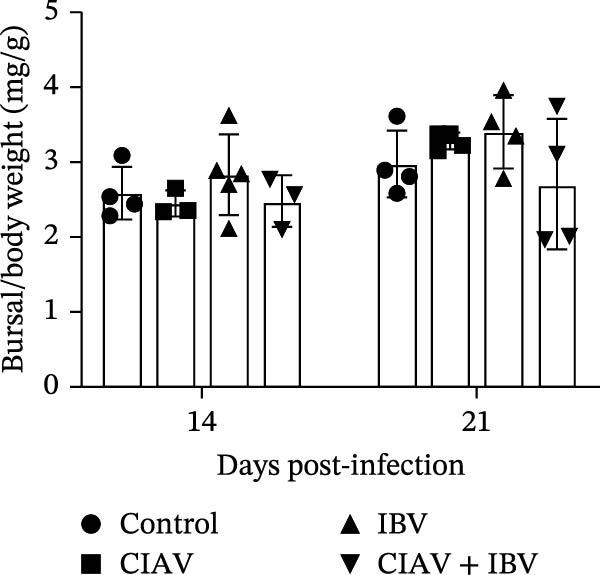
(C)

### 3.3. Compared With IBV Monoinfection, Coinfection of CIAV and IBV Exacerbates Anemia

Hematological parameters were analyzed from blood samples collected at 14 and 21 days postinfection (dpi) to evaluate the impact of CIAV and IBV coinfection (Figure 3). Both the CIAV monoinfected and coinfected groups exhibited significant reductions in HCT and RBC counts compared to the control and IBV monoinfected groups. Notably, no significant difference was observed between the coinfected and CIAV monoinfected groups, indicating that IBV coinfection does not exacerbate CIAV‐induced anemia. In contrast, analysis of leukocyte parameters revealed that the WBC count was significantly lower in the coinfected group than in the IBV monoinfected group. No other significant differences in WBC counts were detected among the remaining groups. These results demonstrate that CIAV infection is the primary driver of anemia in SPF chickens, as evidenced by decreased HCT and RBC levels, while IBV monoinfection had no significant effect on erythrocytes. Although coinfection did not further aggravate anemia, it led to the most pronounced reduction in WBC count, suggesting a potential synergistic suppressive effect on the immune system.

Figure 3Effects of coinfection on white blood cells, red blood cells, and hematocrit in SPF chickens. (A) Hematocrit. (B) White blood cell count. (C) Red blood cell count. Data are shown as mean ± SD. Significant differences between groups were determined by one‐way ANOVA.  ^∗^ *p* < 0.05 (significant);  ^∗∗^ *p* < 0.01 (highly significant).

**Figure:**
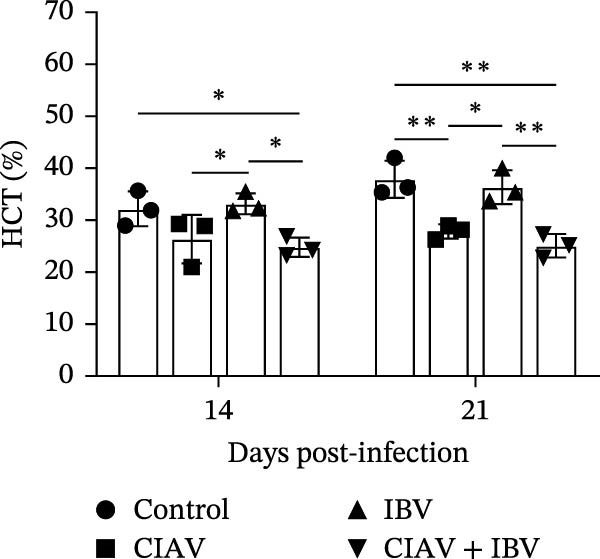
(A)

**Figure:**
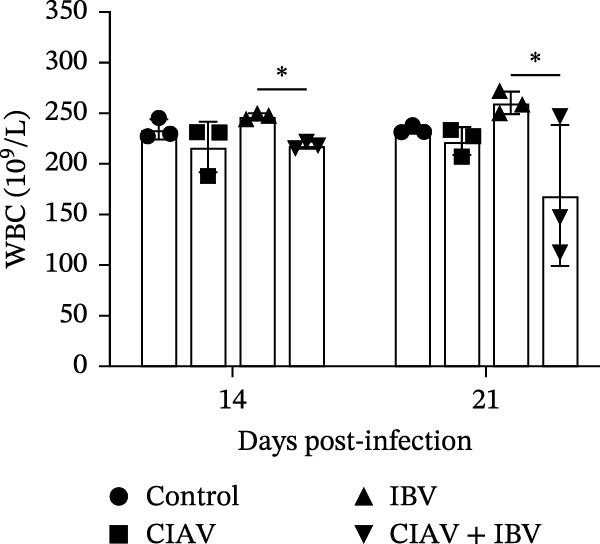
(B)

**Figure:**
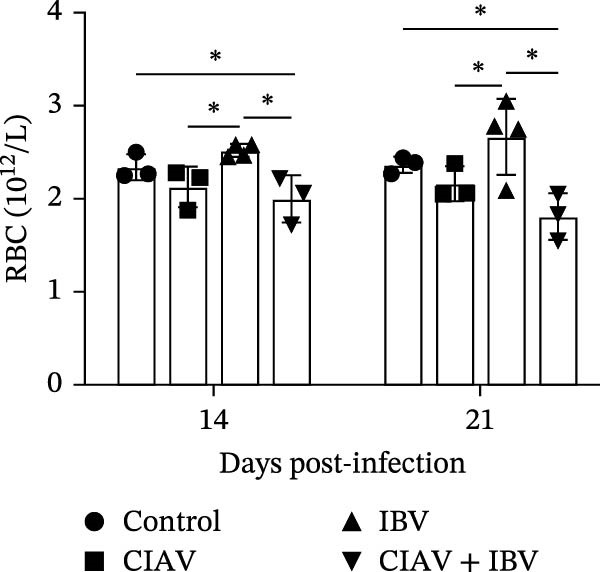
(C)

### 3.4. CIAV Facilitates the Replication of IBV in Tracheal and Pulmonary Tissues

**Figure 4:**
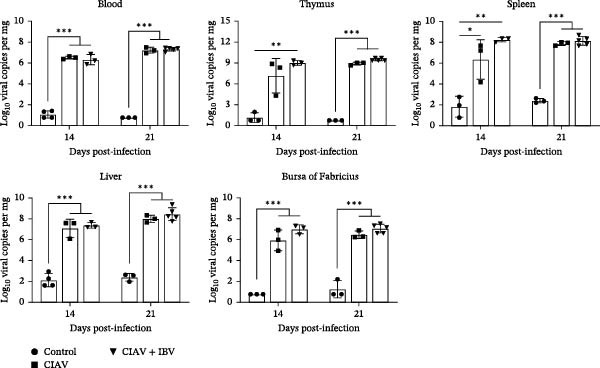
Impact of IBV coinfection on CIAV replication in various tissues of SPF chickens. Data are shown as mean ± SD. Significant differences between groups were determined by one‐way ANOVA.  ^∗^ *p* < 0.05 (significant);  ^∗∗^ *p* < 0.01 (highly significant);  ^∗∗∗^ *p* < 0.001 (extremely significant).

**Figure 5:**
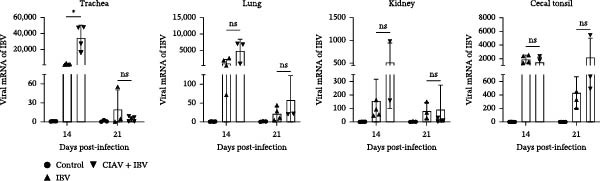
Impact of CIAV coinfection on IBV replication in various tissues of SPF chickens. Data are shown as mean ± SD. Significant differences between groups were determined by one‐way ANOVA.  ^∗^ *p* < 0.05 (significant); ns, no significant.

To investigate the reciprocal impact of coinfection on viral replication dynamics, we quantified CIAV DNA loads in blood, liver, spleen, thymus, and bursa of Fabricius, as well as IBV RNA loads in trachea, lung, kidney, and cecal tonsils across control, monoinfected, and coinfected groups at 14 and 21 days postinfection (dpi) (Figures 4 and 5). Both the CIAV monoinfected and coinfected groups exhibited significantly higher viral loads in all tested tissues compared to the control group at 14 and 21 days postinfection (dpi). But no significant differences in CIAV load were observed between the CIAV monoinfected and coinfected groups in any tissue or at any time point (Figure 4), indicating that IBV coinfection does not alter CIAV replication dynamics. Conversely, IBV replication demonstrated tissue‐specific enhancement under CIAV coinfection. At 14 dpi, the IBV load in the trachea, lung, and kidney of the coinfected group was significantly higher than that in the IBV monoinfected group. By 21 dpi, while tracheal and pulmonary loads decreased to comparable levels in both groups, cecal tonsil loads remained significantly elevated in coinfected chickens. These findings demonstrate that CIAV coinfection significantly influences IBV replication, promoting it in specific tissues. Viral shedding was assessed via oropharyngeal and cloacal swabs (Table S1). The coinfection group demonstrated a 100% detection rate for both viruses in both swab types at all time points. Notably, coinfection increased the detection rate of CIAV in oropharyngeal swabs and of IBV in cloacal swabs from 75% to 100% at 14 dpi. Our results thus demonstrate a clear, one‐way synergistic interaction. Although IBV does not influence CIAV, the presence of CIAV enhances IBV replication in key tissues, prolongs viral persistence, and facilitates more efficient shedding. These effects collectively suggest a significantly elevated transmission risk in commercial poultry settings.

### 3.5. CIAV and IBV Coinfection Leads to Severe Higtopathological Damage

**Figure 6:**
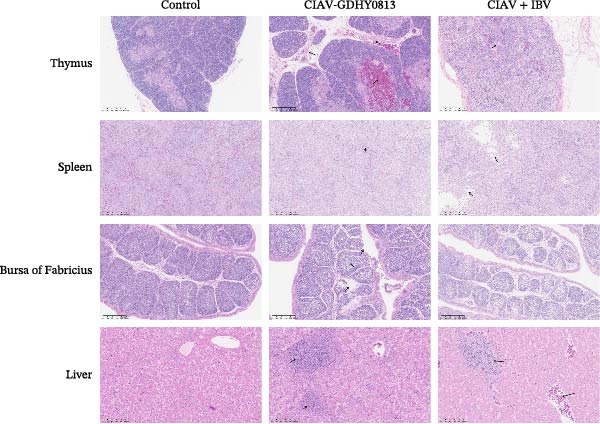
Histopathological changes in the thymus, spleen, Bursa of Fabricius, and liver following coinfection. Representative images are shown at magnifications of ×100 (thymus and spleen) and ×200 (bursa of Fabricius and liver). Lesions are marked with a black arrow. Effect of coinfection on histopathological changes in the thymus, spleen, bursa of Fabricius, and liver of SPF chickens.

**Figure 7:**
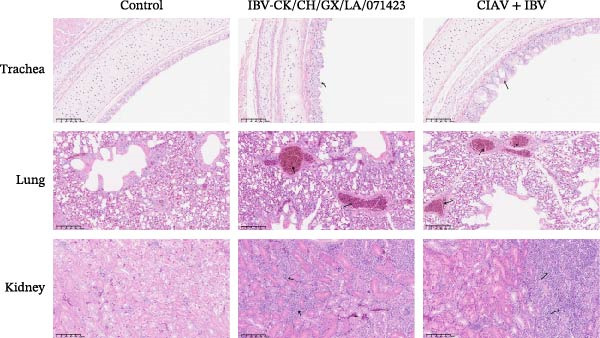
Histopathological changes in the trachea, lung, and kidney following coinfection. Representative images are shown at magnifications of ×200. Lesions are marked with a black arrow. Effect of coinfection on histopathological changes in the trachea, lung, and kidney of SPF chickens.

Histopathological examination was conducted to assess tissue damage in the thymus, spleen, bursa of Fabricius, liver, trachea, lung, and kidney, comparing lesions across different infection groups (Figures 6 and 7).

Chickens in the CIAV monoinfected group exhibited characteristic lesions, including thymic atrophy with focal medullary hemorrhage and widened interlobular septa; blurred demarcation between the splenic red and white pulp accompanied by a reduction in red pulp; mild follicular atrophy and lymphocyte depletion in the bursa of Fabricius; and interstitial hemorrhage with extensive vacuolar degeneration in the liver. In stark contrast, the coinfected group demonstrated markedly exacerbated pathology in these organs. The thymus showed severe atrophy, capsular shrinkage, complete loss of the corticomedullary junction, and the presence of cystic thymic bodies. The spleen exhibited extensive lymphocyte depletion. The bursa of Fabricius presented with aggravated follicular atrophy and significantly widened interfollicular spaces. The liver displayed more severe hemorrhage.

In the coinfected group, the lesions in the respiratory tract and kidney were more severe or distinct. The trachea showed not only ciliary loss but also hypertrophy of goblet cells with cytoplasmic vacuolation. While the severity of pulmonary lesions was comparable between the IBV monoinfected and coinfected groups, a striking difference was observed in the kidneys. IBV monoinfection typically causes renal swelling and focal hemorrhage. However, in the presence of CIAV coinfection, the kidneys demonstrated substantially more extensive and severe hemorrhagic lesions.

### 3.6. Serum Cytokine Responses Following CIAV and IBV Coinfection

**Figure 8:**
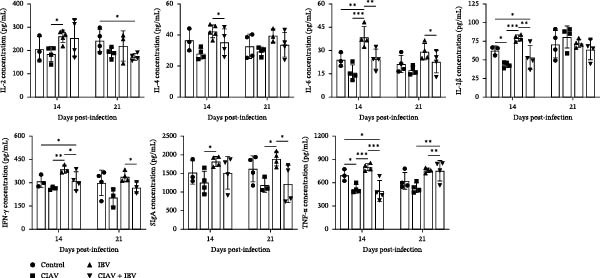
Serum cytokine and sIgA levels measured by ELISA. Serum levels of IL‐2, IL‐4, IL‐6, IL‐1β, IFN‐γ, sIgA, and TNF‐α were detected by ELISA. Data are shown as mean ± SD. Significant differences between groups were determined by one‐way ANOVA.  ^∗^ *p* < 0.05 (significant);  ^∗∗^ *p* < 0.01 (highly significant),  ^∗∗∗^ *p* < 0.001 (extremely significant).

To assess the immunomodulatory effects of CIAV and IBV, serum levels of cytokines (IL‐2, IL‐4, IL‐6, IL‐1β, IFN‐γ, and TNF‐α) and secretory IgA (sIgA) were measured at 14 and 21 dpi (Figure 8). The serum cytokine and sIgA revealed distinct immunomodulatory effects of each virus. IBV monoinfection significantly upregulated a broad spectrum of cytokines at both 14 and 21 dpi. The IBV monoinfected group exhibited significantly higher levels of IL‐2, IL‐4, IL‐6, IL‐1β, IFN‐γ, and sIgA than the CIAV monoinfected group. Conversely, CIAV monoinfection generally suppressed these mediators, with levels consistently below those of the control and IBV groups across time points. The coinfected group demonstrated an intermediate immunologic phenotype. While levels of IL‐2, IL‐4, IL‐6, IL‐1β, IFN‐γ, and sIgA were generally lower than in the IBV group, they displayed dynamic changes. A notable temporal shift occurred in TNF‐α, which was low at 14 dpi (comparable to the CIAV group) but became the highest among all groups by 21 dpi. Of note, IFN‐γ and sIgA levels in coinfected chickens remained low throughout the study period. This pattern highlights the time‐dependent and complex immunomodulatory interaction during coinfection.

## 4. Discussion

As a primary immunosuppressive agent, CIAV inflicts damage on the immune system, thereby predisposing infected birds to secondary or mixed infections. A substantial body of evidence has documented that the immunosuppressive state induced by CIAV can potentiate the pathogenicity of diverse avian viruses, leading to frequent clinical coinfections with pathogens such as IBDV, ALV, MDV, FAdV, and GyH1 [33–39]. Consistent with this, our epidemiological surveillance in Southern China, along with reports from other countries [35, 40], has frequently identified coinfection of CIAV and IBV. In clinical, such coinfections are associated with more severe pathological manifestations and higher mortality rates than single infections. However, despite its clinical relevance, the synergistic pathogenic mechanisms underlying CIAV and IBV coinfection remain poorly defined. In this study, we systematically elucidate the significant synergistic pathogenic effects triggered by coinfection with CIAV and IBV, utilizing prevalent strains from Southern China.

Our experimental data provide conclusive evidence that CIAV and IBV coinfection results in significantly exacerbated clinical disease compared to either monoinfection. This synergistic pathogenicity was quantitatively demonstrated by severe growth retardation, a substantial decline in survival rate (plummeting to 76% in coinfected chickens, compared to 93.5% and 96% in the CIAV and IBV monoinfected groups, respectively), and more pronounced histopathological lesions in central immune organs. The liver and kidney are important organs for compensating anemia caused by CIAV. Coinfected chickens showed enhanced hemorrhage in the liver and kidney without overt parenchymal cell damage. These histopathological changes may be a compensatory regenerative response, rather than just direct viral inflammation. As the primary site of hematopoiesis, the bone marrow is the principal target of CIAV. Consequently, bone marrow histopathology would be indispensable to directly confirm the postulated CIAV‐induced suppression and to conclusively link it to the systemic compensatory reaction observed in the liver and kidney. The absence of such bone marrow analysis in this study is a limitation that clearly defines a critical direction for future research. We observed aggravated atrophy of the thymus and bursa of Fabricius, alongside splenomegaly. These findings align with some reports from Japan and Egypt that described more severe clinical outcomes in cases of CIAV–IBV coinfection [35, 40]. Furthermore, this phenomenon, wherein an immunosuppressive virus like CIAV exacerbates the disease caused by a concurrent pathogen, is not unique to the CIAV–IBV interaction. Similar synergistic effects have been documented in coinfections involving CIAV and other viruses, such as GyH1, MDV, and ALV‐J [30, 36, 41]. This recurrent phenomenon across distinct viral pairs underscores that the enhancement of copathogen virulence by CIAV is a consistent feature of its pathogenesis.

Viral coinfections can significantly alter the biological dynamics of the involved pathogens, including their replication kinetics and tissue tropism—potentiating more severe disease outcomes in hosts [42]. Our study revealed a distinct asymmetric interaction between CIAV and IBV, wherein coinfection significantly enhanced IBV replication in respiratory and lymphoid tissues, yet exerted no notable effect on CIAV replication dynamics. This pattern of one‐way enhancement, whereby CIAV potentiates a coinfecting virus without reciprocal effects, is consistent with previous studies. It has been reported that CIAV enhances the replication of both H9N2 avian influenza virus and the IBV‐Eg/15170F‐SP1 strain across various tissues [40]. This suggests that the enhancement of IBV replication by CIAV may be an inherent feature of coinfection, independent of the specific IBV strain involved. This phenomenon is further corroborated by studies on CIAV and ALV‐J coinfection, where CIAV promoted ALV‐J replication without a corresponding increase in its own viral load [32]. This stands in stark contrast to the mutual enhancement of replication observed in other immunosuppressive virus pairs, such as between CIAV and GyH1 or between MDV and ALV‐J [30, 43]. We posit that this virus‐specific outcome is largely dictated by fundamental viral characteristics, particularly tissue tropism. Unlike the immunosuppressive viruses MDV and ALV‐J, IBV principally targets respiratory and urogenital epithelial cells [44]. This means that IBV is unlikely to directly compete with CIAV within lymphocytes, thereby avoiding any hindrance to CIAV replication. This interpretation is further corroborated by our hematological findings; the absence of exacerbated anemia in coinfected birds indicates that IBV does not directly contribute to or amplify the hematopoietic pathology instigated by CIAV. We hypothesize that the underlying mechanism for this synergy may involve CIAV‐mediated immunosuppression creating a favorable environment for IBV replication. This notion is supported by the significant modulation of serum immune factors such as IFN‐*γ* observed in coinfected chickens (Figure 8). This suggests that CIAV may inhibit the host’s innate immune response, particularly interferon production, thereby delaying or impairing the effective clearance of IBV and indirectly facilitating its replication and pathogenicity [45, 46]. Alternatively, direct competition for host cell resources or indirect interactions mediated through the modulation of different host factors could represent additional mechanisms, both of which warrant further investigation.

However, it should be noted that our sequential infection model, while clinically relevant, examines one specific temporal interaction. Given that CIAV can be transmitted both vertically and horizontally, the pathogenic outcomes might differ under simultaneous infection or if the IBV challenge precedes CIAV. Nevertheless, our model successfully recapitulates a critical and prevalent field scenario, providing a robust experimental foundation for understanding how established CIAV‐induced immunosuppression can alter the outcome of a secondary IBV infection. Furthermore, the current evidence does not fully establish that a functional immunosuppressed state was definitively induced by CIAV in this model. While our data are consistent with the proposed mechanism, the precise immunological drivers of this synergy remain to be fully elucidated. Therefore, a definitive functional assessment, such as quantitative analysis of lymphocyte subsets (CD4^+^ and CD8^+^ T cells) in affected tissues, represents a critical next step. This assessment is essential both to confirm the establishment of immunosuppression and to elucidate the key molecular mechanisms by which CIAV predisposes hosts to secondary infections.

Current vaccination strategies against both pathogens face substantial challenges. For CIAV, no globally licensed therapeutic interventions exist. Although several vaccine candidates have shown promising immunogenicity in experimental settings, live‐attenuated vaccines remain the most widely used option internationally, despite their inherent risk of virulence reversion [4]. China currently lacks access to safe and reliable commercial CIAV vaccines, leaving flocks vulnerable. The situation for IBV is equally complex. The overuse of some vaccine strains not only fails to provide effective protection but also promotes the emergence of new recombinant viruses as gene donors [19], which undoubtedly exacerbates the complexity of IBV epidemics and increases the risk of coinfection with CIAV. In this challenging landscape, the establishment and rigorous enforcement of robust biosecurity protocols, coupled with scientific management, constitute the most fundamental and immediate measures to disrupt viral transmission chains and mitigate coinfections. While indispensable, these biosecurity measures alone are not a panacea. Vaccination remains the cornerstone of sustainable, long‐term infectious disease prevention. Consequently, future research must pivot towards the development of vaccines that are both safer and more efficacious. Vectored vaccines represent a particularly promising avenue. Their capacity to simultaneously elicit robust humoral and cellular immune responses, combined with the potential to deliver protective antigens against multiple pathogens like CIAV and IBV, positions them as an ideal strategy to address coinfection [21]. The systematic exploration of novel viral or bacterial vectors thus presents a prospective pathway for developing innovative and powerful solutions to curb the threat posed by CIAV and IBV coinfection.

In conclusion, this study, using a prevalent CIAV strain and a QX‐genotype IBV strain from Southern China, confirms that coinfection with these two viruses induces significant synergistic pathogenicity in SPF chickens. This effect is primarily characterized by exacerbated clinical disease, increased mortality, and enhanced IBV replication and shedding. These findings reveal the pivotal role of CIAV in viral coinfections and underscore the importance of implementing integrated control strategies against both CIAV and IBV in poultry farms. However, given the considerable genetic diversity of both viruses—particularly the rapid recombination and mutation of IBV—the universality of this synergistic pathogenic effect among different predominant strains requires further investigation. Future research should not only focus on elucidating the specific molecular mechanisms by which CIAV modulates host immunity to facilitate IBV replication but also investigate whether this synergy extends to a broader range of circulating strains.

## Funding

The project is supported by the Guangxi Agriculture Research System Innovation Team (Grant nycytxgxcxtd‐2024‐19), the National Natural Science Foundation of China (Grant 32260876), and the Guangxi Science and Technology Program (Grant GUIKE AD23049005).

## Ethics Statement

All animal experiments were approved by the Animal Ethics Committee of Guangxi University (Approval Number GXU‐2023‐0033) and performed in strict compliance with the institution’s guidelines on animal welfare.

## Conflicts of Interest

The authors declare no conflicts of interest.

## Supporting Information

Additional supporting information can be found online in the Supporting Information section.

## Supporting information


**Supporting Information** Table S1: Viral shedding of CIAV and IBV in oropharyngeal and cloacal swabs.

## Data Availability

The data that support the findings of this study are available from the corresponding author upon reasonable request.
