# Supplementary material for: Chicken Infectious Anemia Virus Markedly Enhances the Pathogenicity of Infectious Bronchitis Virus–Infected Chickens
Source: Transbound Emerg Dis. 2026 Apr 17;2026:2499058. doi: 10.1155/tbed/2499058 (PMC13087508; doi:10.1155/tbed/2499058)
Supplement: Supplementary file 1 — Supporting Information Table S1: Viral shedding of CIAV and IBV in oropharyngeal and cloacal swabs. [file TBED-2026-2499058-s001.doc]

**Supplementary material**

Supplementary Table S1 Viral shedding of CIAV and IBV in oropharyngeal and cloacal swabs.

| Groups | CIAV | | | | IBV | | | |
| --- | --- | --- | --- | --- | --- | --- | --- | --- |
|  | Oropharyngeal swabs | | Cloacal swabs | | Oropharyngeal swabs | | Cloacal swabs | |
|  | 14 d | 21 d | 14 d | 21 d | 14 d | 21 d | 14 d | 21 d |
| Control | 0/4 | 0/4 | 0/4 | 0/4 | 0/4 | 0/4 | 0/4 | 0/4 |
| CIAV | 3/4 | 4/4 | 4/4 | 4/4 | 4/4 | 0/4 | 0/4 | 0/4 |
| IBV | 0/4 | 0/4 | 0/4 | 0/4 | 0/4 | 4/4 | 3/4 | 4/4 |
| CIAV+IBV | 4/4 | 4/4 | 4/4 | 4/4 | 4/4 | 4/4 | 4/4 | 4/4 |
